# Effect of Malnutrition on Femoral Cartilage Thickness in Pediatric Patients

**DOI:** 10.3390/children12081021

**Published:** 2025-08-02

**Authors:** Şükrü Güngör, Raikan Büyükavcı, Fatma İlknur Varol, Emre Gök, Semra Aktürk

**Affiliations:** 1 Department of Pediatric Gastroenterology, Faculty of Medicine, Inonu University, Malatya 44210, Türkiye; sukru.gungor@inonu.edu.tr (Ş.G.); ilknur.varol@inonu.edu.tr (F.İ.V.); emre.gok@inonu.edu.tr (E.G.); 2 Department of Physical Therapy and Rehabilitation, Konya Beyhekim Training and Research Hospital, Konya 42130, Türkiye; 3 Department of Physical Therapy and Rehabilitation, Faculty of Medicine, Inonu University, Malatya 44210, Türkiye; semra.akturk@inonu.edu.tr

**Keywords:** cartilage, child health, malnutrition, ultrasound

## Abstract

**Background/Objectives**: Malnutrition is an imbalance of nutrients required for growth, development, and organ function. Its impact on bone development is known, but its effects on cartilage remain unclear. This study aimed to evaluate the femoral cartilage thickness in children with primary malnutrition. **Methods**: In this cross-sectional observational study, 83 children with primary malnutrition and 62 age- and sex-matched healthy controls were included. Patients with primary malnutrition were classified as mild, moderate and severe. Femoral cartilage thickness measurements of all children were taken by ultrasound from the femoral lateral condyle, femoral medial condyle and intercondylar area for both knees with the patient in a supine position with the knees flexed 90 degrees. **Results**: The right lateral, right medial, left lateral, and left medial femoral cartilages were significantly thicker in patients with malnutrition compared to those without malnutrition (*p* = 0.002, 0.004, <0.001, and 0.001, respectively). A significant negative correlation was found between age, weight Z-score, and height Z-score and triceps skinfold thickness. **Conclusions**: Distal femoral cartilage thickness is significantly greater in children with primary malnutrition. This demonstrates the effect of nutritional factors on cartilage tissue and suggests that children with chronic malnutrition are at risk for both knee joint problems and short stature later in life.

## 1. Introduction

Malnutrition is an imbalance of nutrients required for growth, development, and organ function [1]. There are two types of malnutrition: primary and secondary. Primary malnutrition occurs as a result of inadequate dietary intake, while secondary malnutrition develops as a result of underlying medical conditions such as heart disease, immune deficiencies, renal failure, and celiac disease [2]. Primary malnutrition is common in developing countries as a result of inadequate food supply caused by social, economic and environmental factors [3]. In cases of malnutrition, the body attempts to provide the necessary energy by catabolizing protein, carbohydrates, and fat to protect vital organs. If this adaptive metabolic response is prolonged, all organs except the brain begin to shrink. Growth retardation (due to chronic malnutrition) is defined as a height-for-age Z-score below −2, indicating stunted linear growth due to long-term nutritional deficiency or illness. Acute malnutrition (wasting) is characterized by a weight-for-height Z-score below −2, reflecting recent or severe weight loss. Underweight is defined as a weight-for-age Z-score below −2, which may represent a combination of chronic and acute malnutrition [4,5].

Measuring femoral cartilage thickness by ultrasonography (USG) in the pediatric population has many advantages: it is safe, does not involve ionizing radiation, is noninvasive, is well accepted by children, does not require sedation, and can be performed at the bedside. Therefore, studies evaluating femoral cartilage thickness using USG have gained increasing attention due to their potential to detect early cartilage changes. Femoral cartilage thickness is known to vary based on several factors, including age, gender, and physical activity levels, as these influence cartilage metabolism and load-bearing capacity. Additionally, preexisting inflammatory diseases, such as rheumatoid arthritis and psoriatic arthritis, can accelerate cartilage degradation, leading to measurable thinning. Furthermore, nutritional factors like vitamin D levels play a critical role in cartilage health by modulating bone and cartilage metabolism. Understanding these variables is essential for interpreting USG findings accurately and for distinguishing pathological changes from normal physiological variations [6,7,8]. Moreover, femoral cartilage thickness reflects overall cartilage health and integrity. Nutrition-related factors, including protein, calcium, and vitamin D, influence cartilage metabolism. Studies have demonstrated altered cartilage development in children with protein–energy malnutrition, potentially contributing to joint problems later in life [9,10].

To our knowledge, there is no study in the literature that shows the effects of malnutrition on cartilage in pediatric patients. Therefore, the aim of our study was to determine the effect of primary malnutrition on femoral cartilage thickness in both knees.

## 2. Materials and Methods

### 2.1. Study Design and Ethical Approval

This cross-sectional observational study was conducted at the Pediatric Gastroenterology, Hepatology, and Nutrition Clinic. Ethical approval was obtained from the Inonu University Scientific Research and Publication Ethics Committee, Health Sciences Scientific Research Ethics Committee (approval date: 13 December 2022, session number: 20, decision number: 2022/4163). This study was conducted in accordance with the Declaration of Helsinki, and informed consent was obtained from all participants and/or their legal guardians.

### 2.2. Participants

Children aged 2 to 16 years were included in two groups:

• Patients diagnosed with primary malnutrition
• Age- and sex-matched healthy controls (because femoral cartilage thickness varies with age and sex [11], the control group was matched accordingly to reduce confounding).

Primary malnutrition patients were further classified into mild, moderate, and severe groups based on anthropometric Z-scores (weight, height, BMI, and mid-arm circumference).

### 2.3. Inclusion and Exclusion Criteria

Inclusion criteria: (1) children aged 2–16 years, (2) diagnosis of primary malnutrition based on anthropometric Z-scores, and (3) absence of chronic or systemic disease.

Exclusion criteria: Presence of chronic diseases that may cause secondary malnutrition, such as heart failure, chronic liver disease, renal failure, diabetes mellitus, celiac disease, cystic fibrosis, cerebral palsy, or short bowel syndrome. History of knee injections and/or surgery within the past year.

### 2.4. Anthropometric Measurements

All anthropometric measurements were performed on the day of ultrasound examination by trained personnel using standardized protocols.

Height: Measured without shoes or socks using a portable vertical stadiometer, calibrated to the nearest millimeter. Hair accessories were removed before height measurement.

Weight: Measured wearing light clothing (T-shirt and shorts) with a digital electronic scale calibrated to the nearest 0.1 kg.

Body Mass Index (BMI): Calculated as weight (kg) divided by height squared (m^2^).

Mid-Arm Circumference (MAC): The midpoint between the acromion and olecranon was identified, and three separate measurements were taken using a non-stretchable tape measure; the average was recorded [12].

Triceps Skinfold Thickness (TST): Skin and subcutaneous tissue were gently pulled away from the muscle using two fingers. Three measurements were taken at this site using a caliper device, and the average was calculated [12].

### 2.5. Diagnostic Criteria for Primary Malnutrition

Primary malnutrition describes patients with malnutrition due to inadequate intake and no underlying disease. Anthropometric measurement Z scores between −1 and −2 were classified as mild malnutrition, those between −2 and −3 as moderate malnutrition, and those below −3 as severe malnutrition (Table 1). In this study, we determined the degree of malnutrition according to the weight-for-age Z-score [13].

### 2.6. Ultrasonographic Measurement of Femoral Cartilage Thickness

**Figure 1:**
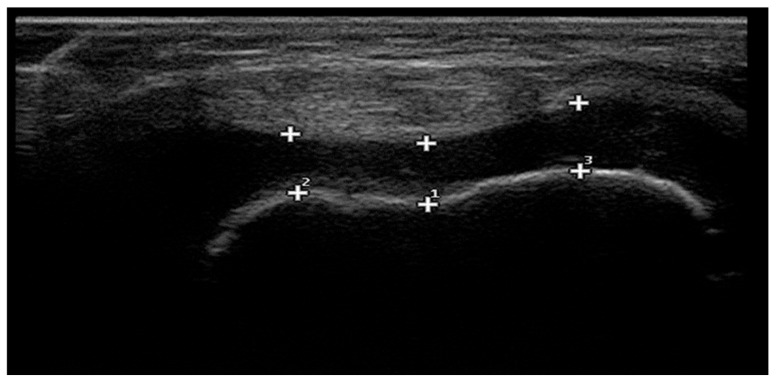
Ultrasound measurement of cartilage thickness. 1: Intercondylar area (ICA), 2: medial femoral condyle (MFC), and 3: lateral femoral condyle (LFC).

Femoral cartilage thickness was measured bilaterally by an experienced physiatrist using an Esaote (Genova, Italy) Mylab 70 ultrasound machine with a 6–18 MHz linear probe. The patient rested in the supine position for 15 min prior to measurement. Measurements were taken with both knees flexed at 90° and cartilage thickness was recorded in millimeters (mm) (Figure 1).

### 2.7. Power Analysis

Using a *t*-test for the difference between two independent means, with an alpha level of 0.05, effect size of 0.5, and power of 0.85, the minimum sample size was calculated as 59 per group (patient and control). The total planned sample size was at least 118.

### 2.8. Statistical Analysis

Data were analyzed using SPSS version 22 (IBM Corp., Armonk, NY, USA). Descriptive statistics are presented as mean ± standard deviation (SD), number (n), and percentage (%). The Kolmogorov–Smirnov test was used to assess the normality of continuous variables. For normally distributed data, comparisons between two groups were made using Student’s *t* tests, and comparisons between two or more groups were made using one-way ANOVA. For non-normally distributed data, the Mann–Whitney U test (two groups) or the Kruskal–Wallis test (multiple groups) were used. Categorical variables were analyzed using Chi-square tests. Differences in cartilage thickness between the healthy control group and the malnutrition group were analyzed using independent Student’s *t* tests. Differences in cartilage thickness according to the severity of malnutrition were analyzed using one-way ANOVA. The correlation between anthropometric measurement Z-scores and cartilage thickness was calculated using the Pearson correlation coefficient. A *p*-value < 0.05 was considered statistically significant.

## 3. Results

The number of cases included in this study was 144. Of these, 83 were malnourished and 61 were healthy controls. The mean age of the malnourished group was 7.84 ± 4.62, and the mean age of the healthy group was 7.86 ± 5.36. The malnourished group included 39 girls (47%) and 44 boys (53%), while the healthy control group included 32 girls (52.5%) and 29 boys (47.5%). There was no significant difference between the groups in terms of age or gender (*p*: 0.992, *p*: 0.512, respectively).

When patients were evaluated according to their weight Z-score, 61 patients (42.4%) were normal, while 16 patients (11.1%) had mild underweight, 43 patients (29.9%) had moderate underweight, and 24 patients (16.7%) had severe underweight. When patients were evaluated according to their height Z-score, 79 patients (54.9%) were normal, 29 patients (20.1%) had mild stunting, 25 patients (17.4%) had moderate stunting, and 11 patients (7.6%) had severe stunting. When patients were evaluated according to the MAC Z-score, 50 patients (34.7%) were normal, 45 patients (31.3%) had mild malnutrition, 28 patients (19.4%) had moderate malnutrition, and 21 patients (14.6%) had severe malnutrition. Accordingly, the MAC Z-score was selected to detect malnutrition of approximately 10% more than the weight loss Z-score (according to Gomez’s malnutrition classification) (Table 1).

There was no significant difference between the groups in terms of age or gender (*p*: 0.976, *p*: 0.516, respectively). We found that the right lateral, right medial, left lateral and left medial cartilages were significantly thicker in malnourished patients than in the healthy group (*p* = 0.002, *p* = 0.004, *p* < 0.001, and *p* < 0.001, respectively). Right and left intermedial cartilage thicknesses were not different between the groups (*p* = 0.174 and *p* = 0.345, respectively) (Table 2).

When cartilage thickness was evaluated according to the degree of malnutrition, we found that the right lateral, right medial, left lateral and left medial cartilages were significantly thicker in patients with moderate to severe malnutrition compared to the healthy group (*p* = 0.002, *p* = 0.004, *p* < 0.001, and *p* < 0.001, respectively). Cartilage was thicker in the mildly malnourished group compared to the healthy control group, but there was no statistically significant difference. Although the right and left intermedial cartilages were thicker in all degrees of malnutrition, this difference was not statistically significant (Table 3).

There was no significant correlation between the BMI Z-score and MAC Z-score and femoral cartilage thickness. We found a significant negative correlation between age, triceps thickness and cartilage thickness in all locations (Table 4).

We found a significant negative correlation between weight Z-score and right lateral, right medial, left lateral and left medial cartilage thicknesses (r = −0.168, *p* = 0.045; r = −0.223, *p* = 0.007; r= −0.209, *p* = 0.012; r = −0.238, *p* = 0.004; respectively). We found a significant negative correlation between height Z-score and right lateral, right medial, left lateral and left medial cartilage thicknesses (r = −0.239, *p* = 0.004; r = −0.257, *p* = 0.002; r = −0.286, *p* = 0.001; r = −0.284, *p* = 0.001; respectively) (Table 4).

## 4. Discussion

Distal femoral cartilage thickness is significantly greater in children with primary malnutrition. This finding demonstrates the effect of nutritional factors on bone and cartilage tissue.

Cartilage formation and degradation is a dynamic process that continues throughout life and is regulated by nutritional, metabolic, and endocrine factors. Femoral cartilage thickness is increasingly being investigated in the evaluation of the musculoskeletal system in children. Spannow et al. provided reference values for normal ultrasonographic measurements in children aged 1–18 years [11]. Prior studies have shown that cartilage thickness is inversely associated with age and skeletal maturity [14,15,16,17]. Although sex differences have been reported in adults—with men having thicker cartilage—studies in pediatric populations have yielded inconsistent results. In our cohort, no significant difference in femoral cartilage thickness was found between sexes.

Femoral cartilage thickness was assessed at three locations in each knee (medial and lateral condyles, and intercondylar area) using USG. Previous studies have shown that measurements from the medial and lateral femoral condyles are more reliable and typically reveal greater thickness at the medial condyle [18,19]. In line with this, we observed significantly greater cartilage thickness in both the medial and lateral femoral condyles in the malnourished group compared to healthy controls.

Moderate to severe malnutrition remains a major public health concern, especially in low- and middle-income countries [20]. Over time, malnutrition leads to multisystem complications, including stunting. While anthropometric parameters are routinely used to assess nutritional status, the musculoskeletal system deserves greater attention in malnourished children. Ultrasonographic reference values for healthy children are valuable for interpreting cartilage changes under pathological conditions [11,14,21]. Interestingly, the increased cartilage thickness we observed—especially in the condylar regions—may seem paradoxical, as cartilage thinning is often linked to degeneration. However, this thickening may reflect delayed endochondral ossification and skeletal maturation, rather than improved cartilage quality.

This interpretation is further supported by the negative correlations found between cartilage thickness and weight-for-age, height-for-age Z-scores, and triceps skinfold thickness—all indicators of poor nutritional status. Chronic undernutrition may result in reduced mechanical loading on joints due to lower body mass, leading to delayed cartilage remodeling [22]. Mid-upper arm circumference (MUAC) did not consistently correlate with cartilage thickness, likely because it predominantly reflects peripheral fat and muscle stores rather than lower limb musculoskeletal development. However, lower MUAC values have been linked to reduced physical performance and muscle function, which may indirectly compromise joint integrity [23].

Comparable findings have been reported in diverse pediatric cohorts. In adolescents, muscle mass inversely correlated with cartilage thickness [24]. Children with cerebral palsy or Down syndrome similarly exhibited negative associations between cartilage thickness and muscle strength or motor performance [7,16]. Furthermore, malnourished children demonstrated reduced diaphragm muscle thickness and function relative to healthy controls [25], underscoring the systemic musculoskeletal impact of malnutrition.

The lack of significant differences in intercondylar cartilage thickness likely reflects biomechanical loading variations. Because the medial and lateral condyles bear the majority of weight during gait, they may be more susceptible to nutritional and mechanical factors. It is crucial to note that thicker cartilage in malnourished children does not equate to superior cartilage health. Cartilage thickness is modulated by growth phase, body composition, muscle mass, mechanical loading, and metabolic status [17,19,24], so thickness alone cannot reliably indicate cartilage integrity. Qualitative parameters—such as matrix composition, collagen architecture, and hydration—may be more clinically informative but are not assessed by thickness measurements alone.

Articular cartilage development is a dynamic process heavily influenced by physical activity, hormonal signals, and nutritional status [26]. Although bone density is genetically influenced, nutrition and exercise during growth are crucial in determining peak musculoskeletal health. Children with inadequate cartilage mineralization and joint support due to malnutrition may be at increased risk of developing musculoskeletal disorders, such as patellofemoral pain syndrome or early-onset osteoarthritis, later in life.

Strengths: This study is one of the few to evaluate the effect of primary malnutrition on femoral cartilage thickness in children using ultrasonography, a non-invasive and reproducible method. The inclusion of a control group matched for age and sex enhanced the internal validity of the results. All ultrasound measurements were performed by a single experienced physiatrist, which minimized interobserver variability.

Limitations: This study has a cross-sectional design, which limits the ability to infer causal relationships. The relatively small sample size may limit the generalizability of the findings. Long-term clinical outcomes related to cartilage changes could not be assessed. BMI Z-scores were excluded from the final analysis, which may have reduced the sensitivity of the nutritional status assessment.

### 4.1. Study Implications and Future Directions

This study highlights the potential impact of malnutrition on articular cartilage development in children. Detecting early cartilage involvement via ultrasonography may provide an opportunity for timely nutritional and rehabilitative interventions. Future longitudinal studies are needed to evaluate whether cartilage thickness normalizes with nutritional rehabilitation and to explore the long-term musculoskeletal consequences of pediatric malnutrition.

### 4.2. Recommendations

Pediatric patients diagnosed with malnutrition should be evaluated not only in terms of anthropometry but also for subclinical musculoskeletal changes. Ultrasonographic assessment of femoral cartilage thickness could be integrated into the nutritional follow-up of children at risk. Multidisciplinary management including pediatricians, nutritionists, and physiatrists may be beneficial for improving both systemic and musculoskeletal outcomes in malnourished children. Future research should include larger and more diverse populations, and evaluate the effect of nutritional interventions on cartilage structure.

## 5. Conclusions

In conclusion, our study shows that cartilage health is affected by the presence of malnutrition, which is one of the major problems of childhood. These findings suggest that malnutrition may affect not only growth and body composition but also joint cartilage development. Ultrasonography appears to be a useful, non-invasive tool to detect early cartilage alterations in pediatric populations at nutritional risk. Early identification and management of such changes could play a role in preventing future joint dysfunction and improving musculoskeletal health outcomes.

## Figures and Tables

**Table 1:** Determination of degree of malnutrition.

	Normal	Malnutrition		
	*n*-%	Mild *n*-%	Moderate *n*-%	Severe *n*-%
Weight Z score	−1 < Weight Z score ≤ 2	−2 < Weight Z score ≤ −1	−3 < Weight Z score ≤ −2	≤−3
	61–42.4	16–11.1	43–29.9	24–16.7
Height Z score	−1 < Height Z score ≤ 2	−2 < Height Z score ≤ −1	−3 < Height Z score ≤ −2	≤−3
	79–54.9	29–20.1	25–17.4	11–7.6
MAC Z score	−1 < MAC Z score ≤ 2	−2 < MAC Z score ≤ −1	−3 < MAC Z score ≤ −2	≤−3
	50–34.7	45–31.3	28–19.4	21–14.6

Abbrevations: MAC: mid-arm circumference.

**Table 2:** Evaluation of differences in cartilage thickness between groups.

	Healthy Group (62) *n*-%	Malnutrition (83) *n*-%	*p*
**Gender**			0.516
Female	32–52.5	39–47	
Male	29–47.5	44–53	
	**Mean ± SD**	**Mean ± SD**	
Age (months)	7.84 ± 4.62	7.86 ± 5.36	0.976
R-FLC (mm)	3.02 ± 0.84	3.64 ± 1.41	0.002
R-ICA (mm)	3.07 ± 0.79	3.26 ± 0.91	0.174
R-MLC (mm)	3.19 ± 0.78	3.73 ± 1.38	0.004
L-FLC (mm)	2.92 ± 0.73	3.59 ± 1.39	<0.001
L-ICA (mm)	3.07 ± 0.76	3.19 ± 0.73	0.345
L-MLC (mm)	3.14 ± 0.77	3.84 ± 1.41	<0.001

Abbreviations: R: right; L: left; FLC: femoral lateral condyle; ICA: intercondylar area; MLC: femoral medial condyle; SD: standard deviation. Statistics: independent Student’s *t* test; Significance level set at *p* < 0.05.

**Table 3:** Evaluation of cartilage thickness according to malnutrition groups.

	Healthy Group (61) Mean ± SD	Mild Malnutrition (16) Mean ± SD	Moderate Malnutrition (43) Mean ± SD	Severe Malnutrition (24) Mean ± SD	*p*
Age (months)	7.84 ± 4.62	4.48 ± 1.08	5.54 ± 0.85	8.14 ± 5.82	0.992
R-FLC (mm)	3.02 ± 0.84 **^α^**	3.59 ± 1.12	3.63 ± 1.53 **^β^**	3.68 ± 1.40 **^β^**	0.034
R-ICA (mm)	3.07 ± 0.79	3.19 ± 0.93	3.24 ± 0.94	3.35 ± 0.56	0.527
R-MLC (mm)	3.19 ± 0.78 **^α^**	3.43 ± 1.22	3.77 ± 1.46 **^β^**	3.87 ± 1.37 **^β^**	0.036
L-FLC (mm)	2.92 ± 0.73 **^α^**	3.56 ± 1.16	3.60 ± 1.41 **^β^**	3.60 ± 1.55 **^β^**	0.010
L-ICA (mm)	3.07 ± 0.76	3.11 ± 0.61	3.15 ± 0.78	3.34 ± 0.73	0.520
L-MLC (mm)	3.14 ± 0.77 **^α^**	3.69 ± 1.34	3.87 ± 1.48 **^β^**	3.90 ± 1.38 **^β^**	0.007

Abbreviations: R: right; L: left; FLC: femoral lateral condyle; ICA: intercondylar area; MLC: femoral medial condyle; SD: standard deviation. Statistics: one way ANOVA, post hoc Scheffe alpha test. A statistically significant difference exists between α and β.

**Table 4:** Correlation of cartilage thickness with age and anthropometric measurements.

		Age	BMI Z-Score	Weight Z-Score	Height Z-Score	MAC Z-Score	TST Z-Score
R-FLC	r	−0.647 **	−0.102	−0.168 **	−0.239 **	−0.071	−0.280 **
	*p*	<0.001	0.230	0.045	0.004	0.398	0.001
R-ICA	r	−0.521 **	0.089	−0.132	−0.134	−0.025	−0.277 **
	*p*	<0.001	0.292	0.115	0.109	0.794	0.001
R-MLC	r	−0.673 **	−0.150	−0.223 **	−0.257 **	−0.055	−0.267 **
	*p*	<0.001	0.076	0.007	0.002	0.516	0.002
L-FLC	r	−0.625 **	−0.116	−0.209 **	−0.286 **	−0.097	−0.284 **
	*p*	<0.001	0.171	0.012	0.001	0.248	0.001
L-ICA	r	−0.531 **	−0.093	−0.121	−0.145	−0.036	−0.268 **
	*p*	<0.001	0.274	0.152	0.083	0.672	0.002
L-MLC	r	−0.650 **	−0.156	−0.238 **	−0.284 **	−0.100	−0.271 **
	*p*	<0.001	0.064	0.004	0.001	0.232	0.002

Abbreviations: R: right; L: left; FLC: femoral lateral condyle; ICA: intercondylar area; MLC: femoral medial condyle; MAC: mid-arm circumference; BMI: body mass index; TST: triceps skinfold thickness: ** Correlation is significant at the 0.001 level.

## Data Availability

The authors declare that the data of this study are stored and can be accessed by contacting the corresponding author (Raikan Büyükavcı) if necessary. The study data are not publicly available due to personal data protection laws in Turkey.

## Abbreviations

The following abbreviations are used in this manuscript:

USG	Ultrasonographic
BMI	Body mass index
MAC	Mid-arm circumference
TST	Triceps skinfold thickness
FLC	Femoral lateral condyle
ICA	Intercondylar area
MLC	Femoral medial condyle

## References

[1] Branca F., Demaio A., Udomkesmalee E., Baker P., Aguayo V.M., Barquera S., Dain K., Keir L., Lartey A., Mugambi G. (2020). A New Nutrition Manifesto for a New Nutrition Reality. Lancet.

[2] Shahrin L., Chisti M.J., Ahmed T., Koletzko B. (2015). Primary and Secondary Malnutrition. Pediatric Nutrition in Practice.

[3] Dipasquale V., Cucinotta U., Romano C. (2020). Acute Malnutrition in Children: Pathophysiology, Clinical Effects and Treatment. Nutrients.

[4] Ozturk M.E., Yabanci Ayhan N. (2023). Evaluation of Malnutrition and Screening Tools in Hospitalized Children. Clin. Nutr. ESPEN.

[5] de Onis M., Onyango A.W., Borghi E., Siyam A., Nishida C., Siekmann J. (2007). Development of a WHO Growth Reference for School-Aged Children and Adolescents. Bull. World Health Organ..

[6] Malas F.U., Kara M., Aktekin L., Ersöz M., Ozcakar L. (2014). Does Vitamin D Affect Femoral Cartilage Thickness? An Ultrasonographic Study. Clin. Rheumatol..

[7] Buyukavci R., Buyukavci M.A., Akturk S., Arslan F.N., Dogan D., Canaloglu S.K. (2021). The Relationship between Motor Performance and Femoral Cartilage Thickness in Children with Down Syndrome. Acta Neurol. Belg..

[8] Ozcakar L., Tunc H., Oken O., Unlu Z., Durmus B., Baysal O., Altay Z., Tok F., Akkaya N., Doğu B. (2014). Femoral Cartilage Thickness Measurements in Healthy Individuals: Learning, Practicing and Publishing with TURK-MUSCULUS. J. Back Musculoskelet. Rehabil..

[9] Gat-Yablonski G., De Luca F. (2017). Effect of Nutrition on Statural Growth. Horm. Res. Paediatr..

[10] Katz J.N., Arant K.R., Loeser R.F. (2021). Diagnosis and Treatment of Hip and Knee Osteoarthritis: A Review. JAMA.

[11] Spannow A.H., Pfeiffer-Jensen M., Andersen N.T., Herlin T., Stenbog E. (2010). Ultrasonographic Measurements of Joint Cartilage Thickness in Healthy Children: Age- and Sex-Related Standard Reference Values. J. Rheumatol..

[12] Puntis J.W.L., Koletzko B. (2008). Clinical Evaluation and Anthropometry. Pediatric Nutrition in Practice.

[13] Khaliq A. (2023). Coexisting Forms of Malnutrition in Children of Pakistan. Ph.D. Thesis.

[14] Windschall D., Trauzeddel R., Haller M., Krumrey-Langkammerer M., Nimtz-Talaska A., Berendes R., Ganser G., Nirschl C., Schoof P., Trauzeddel R.F. (2016). Pediatric Musculoskeletal Ultrasound: Age- and Sex-Related Normal B-Mode Findings of the Knee. Rheumatol. Int..

[15] Pradsgaard D.O., Fiirgaard B., Spannow A.H., Heuck C., Herlin T. (2015). Cartilage Thickness of the Knee Joint in Juvenile Idiopathic Arthritis: Comparative Assessment by Ultrasonography and Magnetic Resonance Imaging. J. Rheumatol..

[16] Adiguzel E., Tok F., Ata E., Yasar E., Yilmaz B. (2018). Ultrasonographic Assessment of Femoral Cartilage Thickness in Patients with Cerebral Palsy. PM R.

[17] Schneider D., Weber R., Nourkami-Tutdibi N., Bous M., Goedicke-Fritz S., Hans M.C., Hein S., Wolf M.A., Landgraeber S., Zemlin M. (2024). Ultrasound-Guided Determination Demonstrates Influence of Age, Sex and Type of Sport on Medial Femoral Condyle Cartilage Thickness in Children and Adolescents. Knee Surg. Sports Traumatol. Arthrosc..

[18] Faber S.C., Eckstein F., Lukasz S., Mühlbauer R., Hohe J., Englmeier K.H., Reiser M. (2001). Gender Differences in Knee Joint Cartilage Thickness, Volume and Articular Surface Areas: Assessment with Quantitative Three-Dimensional MR Imaging. Skelet. Radiol..

[19] Jones G., Ding C., Glisson M., Hynes K., Ma D., Cicuttini F. (2003). Knee Articular Cartilage Development in Children: A Longitudinal Study of the Effect of Sex, Growth, Body Composition, and Physical Activity. Pediatr. Res..

[20] Ssentongo P., Ssentongo A.E., Ba D.M., Ericson J.E., Na M., Gao X., Fronterre C., Chinchilli V.M., Schiff S.J. (2021). Global, Regional and National Epidemiology and Prevalence of Child Stunting, Wasting and Underweight in Low- and Middle-Income Countries, 2006–2018. Sci. Rep..

[21] Sidharthan S., Yau A., Almeida B.A., Shea K.G., Greditzer H.G., Jones K.J., Fabricant P.D. (2021). Patterns of Articular Cartilage Thickness in Pediatric and Adolescent Knees: A Magnetic Resonance Imaging-Based Study. Arthrosc. Sports Med. Rehabil..

[22] Gau C.C., Yao T.C., Gan S.T., Lin S.J., Yeh K.W., Chen L.C., Ou L.S., Lee W.I., Wu C.Y., Huang J.L. (2021). Age, Gender, Height and Weight in Relation to Joint Cartilage Thickness among School-Aged Children from Ultrasonographic Measurement. Pediatr. Rheumatol..

[23] Yaméogo C.W., Cichon B., Fabiansen C., Iuel-Brockdorf A.S., Shepherd S., Filteau S., Traoré A.S., Christensen V.B., Michaelson K.F., Brage S. (2017). Correlates of Physical Activity among Young Children with Moderate Acute Malnutrition. J. Pediatr..

[24] Herrera H.G.A., Llinas P.J., Florez L., Montes C.B., Obando D.V., Solorzano A.D., Loaiza D., Guillen Astete C. (2020). Ultrasound Measurement of Femoral Cartilage Thickness in the Knee of Healthy Young University Students. Rev. Esp. Cir. Ortop. Traumatol..

[25] Sinanoglu M.S., Gungor S., Dag N., Varol F.I., Kenc S., Gok E. (2024). Ultrasound and Shear Wave Elastography Assessment of Diaphragm Thickness and Stiffness in Malnourished Pediatric Patients. Eur. J. Pediatr..

[26] Jones G., Bennell K., Cicuttini F.M. (2003). Effect of Physical Activity on Cartilage Development in Healthy Kids. Br. J. Sports Med..

